# Interplay between Selenium Levels and Replicative Senescence in WI-38 Human Fibroblasts: A Proteomic Approach

**DOI:** 10.3390/antiox7010019

**Published:** 2018-01-20

**Authors:** Ghania Hammad, Yona Legrain, Zahia Touat-Hamici, Stéphane Duhieu, David Cornu, Anne-Laure Bulteau, Laurent Chavatte

**Affiliations:** 1 Unité Propre de Recherche 3404 (UPR3404), Centre de Génétique Moléculaire (CGM), Centre National de la Recherche Scientifique (CNRS), 91198 Gif-sur-Yvette, France; ghania.hammad@cgm.cnrs-gif.fr (G.H.); yonalegrain@gmail.com (Y.L.); ztouat@gmail.com (Z.T.-H.); stephane.duhieu@institut-vision.org (S.D.); 2 Institut de Biologie Intégrative de la Cellule, Commissariat à L’énergie Atomique et aux Énergies alternatives (CEA), Centre National de la Recherche Scientifique (CNRS), Université Paris Sud, Université Paris-Saclay, 91190 Gif-sur-Yvette, France; david.cornu@i2bc.paris-saclay.fr; 3 Institut de Génomique Fonctionelle de Lyon (IGFL), Unité Mixte de Recherche 5242 Centre National de la Recherche Scientifique/Ecole Normale Supérieure de Lyon (CNRS/ENS UMR5242), 69007 Lyon, France; anne-laure.bulteau@ens-lyon.fr; 4 Centre International de Recherche en Infectiologie (CIRI), 69007 Lyon, France; 5 Unité 1111, Institut National de la Santé Et de la Recherche Médicale (INSERM U1111), 69007 Lyon, France; 6 Unité Mixte de Recherche 5308, Centre National de la Recherche Scientifique/Ecole Normale Supérieure de Lyon/Université Claude-Bernard-Lyon-I (CNRS/ENS/UCBL1 UMR5308), 69007 Lyon, France

**Keywords:** proteomics, 2-Dimensional Differential in-Gel Electrophoresis (2D-DIGE), selenium, protein abundance, selenoprotein, replicative senescence, WI-38 cells

## Abstract

Selenoproteins are essential components of antioxidant defense, redox homeostasis, and cell signaling in mammals, where selenium is found in the form of a rare amino acid, selenocysteine. Selenium, which is often limited both in food intake and cell culture media, is a strong regulator of selenoprotein expression and selenoenzyme activity. Aging is a slow, complex, and multifactorial process, resulting in a gradual and irreversible decline of various functions of the body. Several cellular aspects of organismal aging are recapitulated in the replicative senescence of cultured human diploid fibroblasts, such as embryonic lung fibroblast WI-38 cells. We previously reported that the long-term growth of young WI-38 cells with high (supplemented), moderate (control), or low (depleted) concentrations of selenium in the culture medium impacts their replicative lifespan, due to rapid changes in replicative senescence-associated markers and signaling pathways. In order to gain insight into the molecular link between selenium levels and replicative senescence, in the present work, we have applied a quantitative proteomic approach based on 2-Dimensional Differential in-Gel Electrophoresis (2D-DIGE) to the study of young and presenescent cells grown in selenium-supplemented, control, or depleted media. Applying a restrictive cut-off (spot intensity ±50% and a *p* value < 0.05) to the 2D-DIGE analyses revealed 81 differentially expressed protein spots, from which 123 proteins of interest were identified by mass spectrometry. We compared the changes in protein abundance for three different conditions: (i) spots varying between young and presenescent cells, (ii) spots varying in response to selenium concentration in young cells, and (iii) spots varying in response to selenium concentration in presenescent cells. Interestingly, a 72% overlap between the impact of senescence and selenium was observed in our proteomic results, demonstrating a strong interplay between selenium, selenoproteins, and replicative senescence.

## 1. Introduction

Selenium has been shown to be an essential trace element in all animals. Deficiency of selenium has been linked to several diseases and disorders, including an increased risk of cancer and of cardiovascular and neurological diseases, as well as a decrease in immune function [1,2,3]. Most of the beneficial effects of selenium are due to the existence of a pool of selenoproteins, which are involved in redox biology and homeostasis. In effect, the selenoproteins have the ability to translationally incorporate a rare amino acid residue, selenocysteine (Sec, U), using a specific and complex machinery (for details, see [4]). The selenocysteine residue is more reactive and less sensitive to oxidation than its sulfur analog cysteine. At the catalytic site of enzymes, selenocysteine participates in redox reactions, which are essential for antioxidant defense, redox homeostasis, and cell signaling [5,6,7,8]. Gene inactivation in mice of certain selenoproteins, such as glutathione peroxidase 4 (Gpx4), thioredoxin reductase 1 (Txnrd1), or SelenoT, leads to embryonic lethality. Therefore, it is comprehensible that gene inactivation of Sec-tRNA^[Ser]Sec^ or Selenocysteine insertion sequence binding protein-2 SECISBP2 [9,10], altering selenoprotein synthesis, is also fatal in mice. The level of selenoprotein expression in the organism is tightly regulated not only by the bioavailability of selenium, but also by exogenous stimuli [11]. For in vitro cultured cells, the level of selenium dictates the range of selenoprotein expression in accordance with a cell-line-specific hierarchy [4]. The interplay between selenium levels and exogenous stimuli, such as aging or oxidative stress, has only been studied in specific cases [11,12,13,14].

Aging is a slow, complex, and multifactorial process, resulting in a gradual and irreversible decline of various functions of the body, making organisms more vulnerable and more likely to die [11]. Several cellular aspects of organismal aging are recapitulated in the replicative senescence of cultured human diploid fibroblasts, such as embryonic lung fibroblast WI-38 cells, which thus offer a commonly used cellular model to study in vitro features of the aging phenomenon [15]. Cellular senescence, also referred to as replicative senescence, was initially described by Hayflick and Moorehead in 1961 [16]. Senescence is defined by the finding that diploid cells go through a finite number of divisions—also referred to as the Hayflick limit [16]. Cellular senescence is characterized by an irreversible cell cycle arrest via the p53, pRb, p16, and p21 signaling pathways [17,18,19]; telomere shortening; accumulation of oxidative damage; and several senescence-associated markers, including β-galactosidase (SABG) and heterochromatin foci (SAHF). Interestingly, the number of senescent cells increases with age and senescent-associated phenotypes are considered to be predictive features of age-related phenotypes. In various cell types, premature senescence can be induced by exogenous stimuli. For example, repeated exposure to mild oxidative stress (utraviolet radiation (UV) or tert-Butyl hydroperoxide) provokes a stress-induced premature senescence (SIPS) phenotype [20,21,22] in human diploid fibroblasts. Additionally, in cancer cells, the use of drug therapy can also lead to premature senescence, namely therapy-induced senescence (TIS) [23,24]. Although, in every case, a G_0_ cell cycle arrest is observed, in general, the molecular mechanisms involved and intracellular targets differ between the different senescence phenotypes.

Even though redox status is implicated in the aging phenotype, the link between selenium and aging or replicative senescence has, so far, been poorly investigated (reviewed in [11]). Primarily, it was reported that selenium levels and selenium-dependent glutathione peroxidase activity decreased in the serum of healthy Italian subjects [25]. Interestingly, it was observed in this study that the selenium levels in the elderly were much more scattered than those in young persons. It has been suggested that the decrease of selenium status in aging could lead to a weakening of the antioxidant defense and a decrease in longevity, although the molecular mechanisms and selenoproteins involved remain to be identified. At the cellular level, a few studies have reported extension of the replicative lifespan of cultured cells with selenium supplementation [13,26]. In the most recent study, a 30 nM selenium supplementation of the culture medium significantly reduced the level of senescence markers, including signaling molecules (p16, p21, p53), telomere shortening, SABG and SAHF [13]. As a corollary, the expression of several selenoproteins was altered by replicative senescence. In this study, it was found that selenoproteins were regulated at both the transcriptional and translational levels.

In the present study, we have further investigated the interrelation between selenium and replicative senescence at the molecular level using a proteomic approach. We have compared the response to senescence with long-term growth in culture media containing various levels of selenium, previously found to modulate the replicative capacity of WI-38 cells. Due to a large overlap in protein targets, our data favor an action of selenium in the generic replicative senescence pathway, rather than a premature senescence similar to SIPS.

## 2. Materials and Methods

### 2.1. Cell Growth

Human embryonic fibroblasts WI-38 were purchased from SIGMA (Chicago, IL, United States of America) (passage 15, estimated cumulative population doublings (CPD) 30), grown and maintained in 75 cm^2^ plates in Dulbecco’s Modified Eagle Medium (D-MEM) supplemented with 10% fetal calf serum (FCS), 100 µg mL^−1^ streptomycin, 100 UI mL^−1^ penicillin, 1 mM sodium pyruvate, and 2 mM l-glutamine. Cells were cultivated in 5% CO_2_ at 37 °C and a humidified atmosphere. The different culture media referred to as depleted (Dpl), control (Ctl), and supplemented (Sup) were prepared according to [13] and contained, respectively, 3, 15, and 45 nM of selenium, as determined by Inductively coupled plasma-mass spectrometry ICP-MS [12,13,27,28]. The quantification of the selenium levels in the raw FCS (150 nM) was obtained with a limit of quantification (LOQ) of 2.5 nM. Although much lower than selenium levels in serum, the concentration of 45 nM in the culture medium yielded a strong increase in protein expression for several selenoproteins when selenite was used to supplement the medium [13]. When cells reached confluence (approximately at 100,000 cells per cm^2^), they were passaged in a new flask at 10,000 cells per cm^2^ density. WI-38 cells were counted at each passage to calculate the Population Doubling (PD) using the equation ΔPD = log ((Final Cell Number)/(Initial Cell Number))/log (2). Cumulative PD was plotted as a function of time.

### 2.2. Protein Extraction. 2D Clean-Up Kit. Protein Quantification. Verification of Protein Quality by SDS PAGE

All chemical reagents purchased were 2D-DIGE grade (i.e., PlusOne, GEHealthcare, Little Chalfont, United Kingdom). Whole protein extracts were obtained from 75 cm^2^ plates using cell lysis buffer composed of 40 mM Tris-HCl pH 7.8, 8 M urea, 2 M thiourea, 4% (*w/v*) 3-[(3-Cholamidopropyl)dimethylammonio]-1-propanesulfonate hydrate (CHAPS), 50 mM DL-Dithiothreitol DTT, and protease inhibitor (Complete Mini, Roche Diagnostics). Cells were disrupted by passing them through a 23 gauge syringe 10 times. The homogenates were centrifuged at 20,000 g for 1 h at 4 °C. The supernatant was collected and conserved at −20 °C. An aliquot of crude protein extract was precipitated and washed according to the 2D clean-up kit procedure (GEHealthcare). Pellets were resuspended in 50 µL of Urea, Thiourea, CHAPS (UTC) buffer (8 M urea, 2 M thiourea, 4% CHAPS). Protein concentrations were measured using the Detergent compatible (DC)kit protein assay kit (Biorad) in microplate assays (BMG Labtech GmbH, Ortenberg, Germany). To verify the integrity of protein extracts and correct quantification of protein concentrations, 5 µg of each sample was separated in Bis-Tris NuPAGE Novex Midi Gels (Life Technologies, Carlsbad, CA, United States of America, under 3-(N-Morpholino)propanesulfonic acid, 4-Morpholinepropanesulfonic acid (MOPS) conditions. The gel was then stained using Coomassie staining solution.

### 2.3. 2-Dimensional Differential in-Gel Electrophoresis (2D-DIGE) Labeling, Isoelectric Focusing Electrophoresis (IEF), and Sodium Dodecyl Sulfate Polyacrylamide Gel Electrophoresis (SDS-PAGE ) of Protein Samples

**Figure 1:**
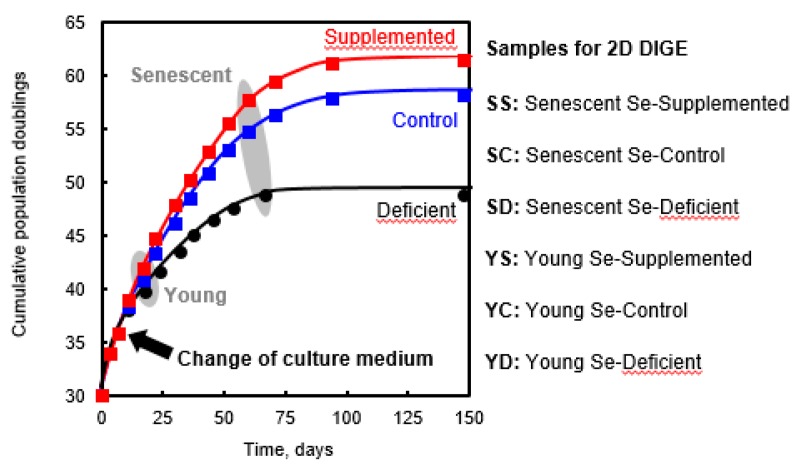
The replicative life span of WI-38 cells is affected by the selenium level of the culture medium. At cumulative population doublings (CPD) 35.8, WI-38 cells were plated in three different conditions, namely, Se-deficient (Dpl, black dots), Se-control (Ctl, blue square), and Se-supplemented (Sup, red square) for two passages and until they reached the senescent stage. The gray ellipses illustrate the time and stage the cells were harvested for 2-Dimensional Differential in-Gel Electrophoresis (2D-DIGE) analyses.

**Figure 2:**
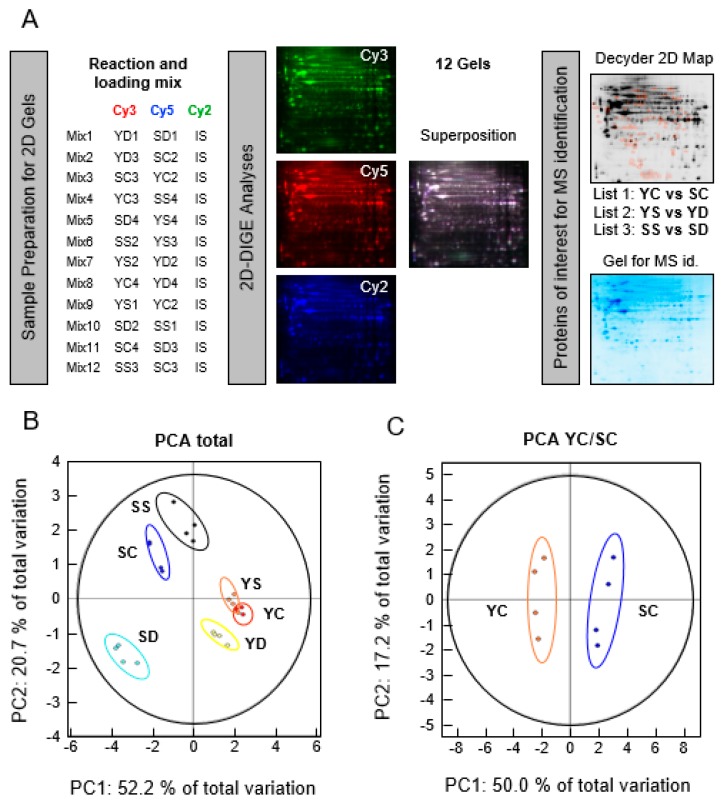
Experimental design and statistical validation of our 2D-DIGE analyses. YD: young Se-depleted; SD: senescent Se-depleted; SC: senescent Se-control; YC: young Se-control; SS: senescent Se-supplemented; YS: young Se-supplemented; MS: mass spectrometry; PCA: principal component analysis; PC1: principal component 1; PC2: principal component 2.

Four experimental replicates for each set of conditions were collected from cells grown in four individual flasks. As shown in Figure 1, six growth conditions were generated, i.e., young Se-depleted (YD), young Se-control (YC), young Se-supplemented (YS), senescent Se-depleted (SD), senescent Se-control (SC), and senescent Se-supplemented (SS). In total, 24 different protein extracts were analyzed using the 2D-DIGE strategy. A quantity of 50 µg of protein from each sample was transferred to a different tube (i.e., 24 in total), and the volume adjusted to 11 µL with UTC buffer. An internal standard (IS) was made by mixing 25 µg of each sample in another tube, with the volume completed to 132 µL with UTC buffer. CyDye DIGE Fluor minimal dyes (GEHealthcare, Little Chalfont, United Kingdom), which react with lysine residues, were resuspended at 400 pM/µL concentration in dimethylformamide (DMF). A quantity of 1 µL of a specific CyDye solution (i.e., Cy3 or Cy5) was add to the 11 µL of protein sample according to the schematic in Figure 2A. Biological replicates were randomly labelled to prevent dye bias. On the other hand, 12 µL of Cy2 was added to the 132 µL of internal standard. The labeling reaction was left to occur for 30 min on ice, protected from light. To the Cy3 and Cy5 reactions, 1 µL of DIGE stop solution (10 mM Lysine) was added. On the other hand, 12 µL of DIGE stop solution was added to the Cy2 (IS) reaction. The stop reaction was left to occur for 10 min protected from light at 4 °C. Twelve mixes composed of one Cy3 (13 µL), one Cy5 (13 µL), and 13 µL of the IS (Cy2) reaction were generated according to the table in Figure 2A. The volume was completed with 311 µL of isoelectric focusing electrophoresis (IEF) rehydration buffer composed of 8 M urea, 2 M thiourea, 2% (*w/v*) CHAPS, 12 µL/mL DeStreak reagent (GEHealthcare, Little Chalfont, United Kingdom), 0.5% (*v/v*) immobilized pH gradient (IPG) Buffer (3–10 non-linear (NL)), and traces of Bromophenol blue. This solution was loaded onto an 18 cm Immobiline DryStrip (3–10 NL) by passive rehydration under a layer of DryStrip Cover Fluid (GEHealthcare, Little Chalfont, United Kingdom) overnight. Then, IEF was performed using IPGPhor2 (GEHealthcare, Little Chalfont, United Kingdom) with the following run: 150 V for 1 h, 200 V for 1 h, gradient 200–1000 V for 9 h, gradient 1000–8000 V for 3 h, and 8000 V until total volt hours (Vh) reached 35,000. At this stage, the strips were kept frozen at −20 °C until further use.

The strips were thawed and incubated for 12 min in equilibration buffer 1 composed of 50 mM Tris-HCl pH 8.6, 6 M urea, 2% (*w/v*) SDS, 30% (*v/v*) glycerol, and 65 mM DTT. Then, the strips were transferred for 12 min to equilibration buffer 2, composed of 50 mM Tris-HCl pH 8.6, 6 M urea, 2% (*w/v*) SDS, 30% (*v/v*) glycerol, and 250 mM Iodoacteamide. After the equilibration stage, each strip was positioned on top of a 12.5% acrylamide (acrylamide/bis acrylamide, 37.5:1) SDS-PAGE and run overnight using the Ettan DALTII system (GEHealthcare, Little Chalfont, United Kingdom), allowing the simultaneous migration of six gels. Migration conditions were as follows: 15 W per gel for 30 min, and 25 W per gel for 16 h, under a water cooling system. It should be noted that the gels were cast in low-fluorescence glass plates to allow a direct reading with a Typhoon fluorescence scanner.

### 2.4. Fluorescence Scanning of the Gel and Decyder Analysis

After the second-dimension electrophoresis run, the plates containing the gels were carefully cleaned and directly scanned with a Typhoon FLA 9500 (GEHealthcare, Little Chalfont, United Kingdom), without uncasting the gel. The orientation of the plates was kept identical for the 12 gels. Every gel was scanned three times with the following parameters: for Cy2, laser 488 nm, emission filter 520BP40, photomultiplier voltage setting (PMT) 490; for Cy3, laser 532 nm, emission filter 580BP30, PMT 520; for Cy3, laser 633 nm, emission filter 670BP30, PMT 470. Importantly, the scanning parameters were kept identical for the 12 gels with a resolution set at 100 µm. The data analysis, including the principal component analysis (PCA), was performed with Decyder 7.0 software (GEHealthcare, Little Chalfont, United Kingdom) according to the manufacturer’s instructions.

### 2.5. Mass Spectrometry Protein Analysis

#### 2.5.1. Sample Preparation

A preparative gel was formed with 500 µg of protein completed with rehydration buffer to a total of 340 µL. Rehydration, migration of the strips, and SDS-PAGE were performed as described earlier for the fluorescence-labeled samples. Each spot of interest was localized in the Coomassie-stained 2D-gel, excised, and subjected to enzymatic digestion. Trypsin digestion of selected spots was performed following reduction/alkylation of cysteine residues as described previously [29] with the addition of 10 µL of trypsin (12.5 ng/µL) per spot and 20 µL of extraction solvent for peptide extraction. Tryptic peptides were vacuum-dried and finally resuspended in 0.1% formic acid and 5% acetonitrile before nanoscale liquid chromatography coupled to tandem mass spectrometry (nanoLC-MS/MS) analyses.

#### 2.5.2. NanoLC-MS/MS Analyses

Tryptic peptides were analyzed by nanoLC-MS/MS analysis using first a nanoAcquity liquid chromatograph (Waters, Milford, MA, United States of America) coupled to a quadrupole-time-of-flight (Q-TOF) Premier mass spectrometer (Waters, Milford, MA, United States of America), and secondly an EASY-nLC II HPLC system (Proxeon, ThermoScientific, Waltham, MA, United States of America) coupled to a nanoESI-Linear Trap Quadropole (LTQ)-Orbitrap Velos mass spectrometer (Thermo Scientific) in case no significant protein identification was obtained in the first analysis. Nano-liquid chromatography elution conditions were similar for both systems, operated with a flow rate of 300 nL/min and an acetonitrile gradient of 5–40% (*v/v*) over 20 min. The nanoLC system (Waters, Milford, MA, United States of America) coupled to the Q-TOF Premier was equipped with a trapping column (C18 symmetry, 180 μm × 20 mm, particle size 5 μm, from Waters) and an analytical column (BEH 130 C18, 75 μm × 100 mm, particle size 1.7 μm, from Waters). In the Q-TOF analysis, MS/MS spectra were acquired by a data-dependent acquisition method involving selection of the three precursors giving the most intense signals. Raw data acquired on the Q-TOF were processed with a ProteinLynx Global Server (Waters). For proteins identified with less than two peptides, the tryptic peptide samples were further analyzed on the more sensitive LTQ-Orbitrap Velos mass spectrometer (Thermo Scientific). In the nanoLC system coupled to the LTQ-Orbitrap, peptide separation was performed on a reversed-phase C18 nanoHPLC column (100 µm inner diameter, 5 µm C18 particles, 15 cm length, NTCC-360/100-5, from NikkyoTechnos (NikkyoTechnos Co., Tokyo, Japan). For the LTQ-Orbitrap, MS/MS spectra were acquired using the data-dependent acquisition mode operating with a Top20 collision induced dissociation (CID) method: the mass of the precursors was measured with high resolution (60,000 full width at half maximum (FWHM)) in the Orbitrap and the twenty most intense ions, above an intensity threshold of 5000 counts, were selected for Collision-Induced Dissociation (CID) fragmentation and analysis in the LTQ. Raw data acquired with the LTQ-Orbitrap were processed with Proteome Discoverer 1.3 software (Thermo Fisher Scientific, Waltham, MA, United States of America).

#### 2.5.3. Protein Identification

Proteins were identified using the MASCOT search engine (Matrix Science, London, UK) against the Swissprot 0112 database with carbamidomethylation of cysteines set as a fixed modification, and oxidation of methionines as variable modifications. Peptide and fragment mass tolerances were set at 20 ppm and 0.1 Da, respectively, for Q-TOF data, and at 5 ppm and 0.6 Da, respectively, for Orbitrap data. Only proteins identified with at least two peptides with a score higher than the identity threshold calculated at a false discovery rate of less than 1% (Mascot decoy option) were considered. The generated protein lists were analyzed with the use of Ingenuity Pathways Analysis™ software (QIAGEN Inc., Available Online: https://www.qiagenbioinformatics.com/).

## 3. Results

### 3.1. 2-Dimensional Differential in-Gel Electrophoresis (2D-DIGE) and Mass Spectrometry Identification of Protein Spots

Replicative senescence is characterized by a finite number of cell divisions under defined growth conditions. We have recently shown that selenium levels in the culture medium modulated the proliferative capacity of WI-38 [13]. In other words, selenium supplementation (Sup) significantly increased the number of cell divisions and decreased the senescence-associated markers and phenotypes, in comparison with control (Ctl) conditions. On the other hand, selenium deficiency (Dpl) accelerated entry into senescence and reduced the proliferative capacity of WI-38 cells, measured by the population doubling at each passage. This acceleration of the senescence-associated phenotype coincides with the decrease in selenoproteins involved in antioxidant defense, glutathione peroxidase 1 (Gpx1) and glutathione peroxidase 4 (Gpx4). Experimentally, at CPD 35.8, mitotically active WI-38 cells, initially grown in Ctl medium, were plated in three different conditions, namely Dpl, Ctl, and Sup. After two passages, young WI-38 cells grown in the different culture media were harvested, at CPD 39.7, 40.4, and 41.9, respectively, from four experimental replicates, and referred to as Young Dpl, Ctl, and Sup (YD, YC, and YS, respectively). As previously observed, even after two passages of young cells in various selenium-containing media, a difference of cell proliferation could be noticed. In parallel, additional flasks were maintained in respective media and harvested (again in four experimental replicates) when they reached presenescent stage, at CPD 48.7, 54.7, and 57.6, respectively, and were referred to as senescent Dpl, Ctl, and Sup (SD, SC, and SS, respectively), see Figure 1.

A proteomic study was then applied to our samples. Comparison of expression patterns between our six different cell growth conditions was performed using 2D-DIGE methodology (Figure 2A), which displays additional advantages compared with classical 2D-gel proteomic analyses. We took advantage of the high resolution of the 2D-gel electrophoresis that allows the separation of proteins as spots according to their isoelectric point (pI) in the first dimension, and their molecular weight in the second dimension. This technique is particularly efficient to separate protein isoforms with different posttranslational modifications (PTMs). The CyDye fluorescent minimal labeling provides at least the same sensitivity as protein silver staining, but with a much higher dynamic range for quantification. Usually, 2500 protein spots can be mapped in a typical 2D-gel from a whole cell extract. An important feature of the 2D-DIGE methodology is the use of an internal standard in all gels, which allowed a reliable spot assignment between the 12 different gels and a precise inter-gel spot quantification. The scanned images were analyzed with Decyder 7.0 software (GEHealthcare). Six groups, namely YD, YC, YS, SD, SC, and SS were assigned to the various scans, each group being composed of four replicates. The gel images were treated with an exclusion volume filter of 30,000 to remove artifacts from the analyses. A master gel was identified by the software, with 2499 spots. Among this number of spots in the master gel, 1155 spots were found in 80% of the gel maps (i.e., 32 out of 36), and were used for further analyses. In order to validate the quality of the replicates, and, if necessary, remove an outlier replicate from the analysis, we performed a principal component analysis (PCA). In effect, when we applied a statistical filter to the spot maps (one-way analysis of variance (ANOVA) < 0.01), we were able to cluster the 2D protein profiles into six groups of four replicates (Figure 2B) corresponding to our six experimental conditions. The first component separated the protein profiles according to the number of passages (young vs. senescent cells, principal component 1 (PC1): 52% of total variation), while the second dimension differentiated the sample according to the selenium variation (Dpl, Ctl vs. Sup, principal component 2 (PC2): 20.7% of total variation). This statistical analysis indicated that protein expression was more impacted by senescence phenotype than by selenium variation, which seemed to be more subtle. In addition, it appeared that SD, SC, and SS were much more segregated than YD, YC, and YS, indicating that the selenium levels of the culture medium induced more differences in protein expression in senescent than in young cells. When considering only young and senescent cells grown in control medium, YC and SC, respectively, the segregation between the two groups was also obvious with PCA (Figure 2C).

**Figure 3:**
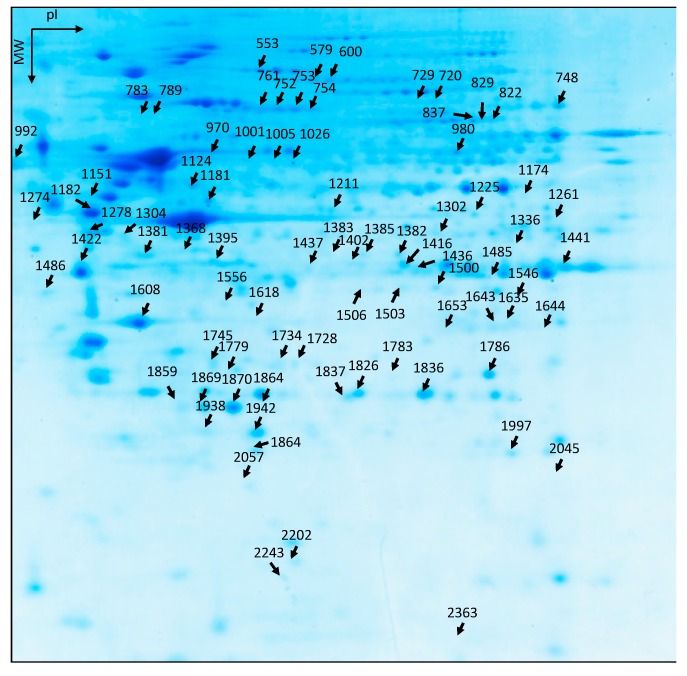
Spot position of the proteins of interest in the spot map of a blue-stained preparative 2D-gel. A quantity of 500 µg of protein sample was separated on a 2D-gel. The identified spots were excised and sent for mass spectrometry identification.

**Figure 4:**
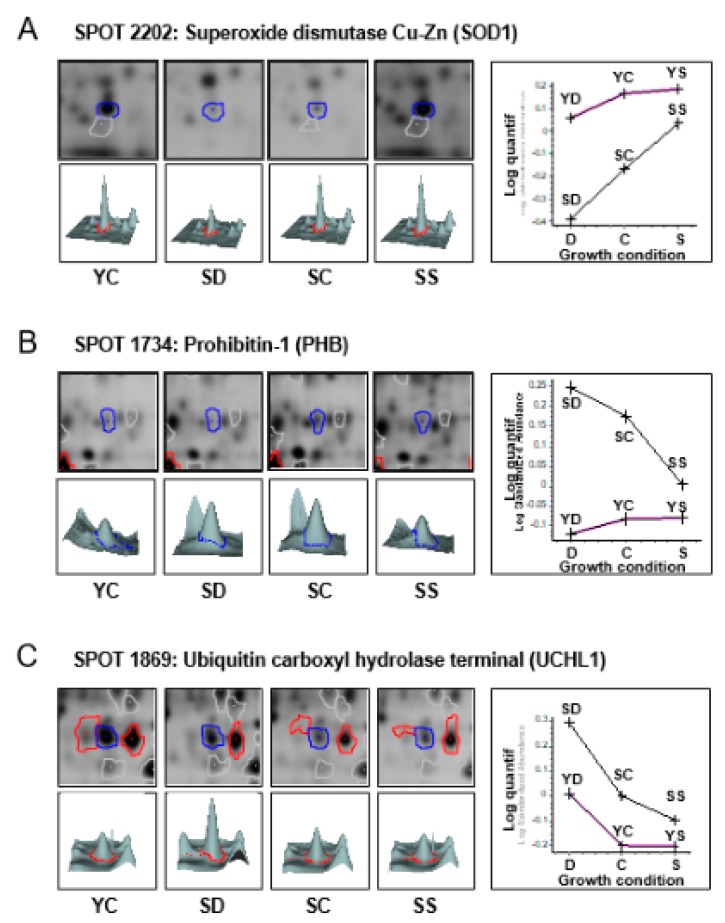
Typical examples of 2D spots differentially regulated by senescence and selenium levels of the culture media. Respective spot maps in 2D (top panels) and 3D (bottom panel) and average quantification (right graphs) are shown for spots number 2202 (**A**), 1734 (**B**), and 1869 (**C**).

When we applied specific filters for differentially and significantly expressed proteins (±50% and *t*-test < 0.05), we found 136 spots among the 1155 common to 80% of the spot maps. In other words, the expression of almost 12% of the proteome was affected by either replicative senescence and/or selenium levels of the culture media. In the preparative gel stained with Coomassie staining, only 81 spots were noticeable (see Figure 3). These protein spots were collected and sent for mass spectrometry protein identification and peptide match search with the human proteome databank as described in Materials and Methods. Basically, protein identification was performed after trypsin digestion with a Q-TOF mass spectrometer, except when two or less peptides were identified by MS/MS. In these cases, the remaining tryptic digestion was analyzed with an Orbitrap mass spectrometer, which provided more peptide sequences than the Q-TOF analysis. Among the 81 spots, 123 proteins were identified. The list of identified proteins together with the mass spectrometer used are indicated in Table 1. Three lists of proteins of interest were generated: (i) proteins differentially regulated between YC and SC (±50% and *t*-test < 0.05, see Table S1); (ii) proteins differentially regulated between YD and YS (same cut-off parameters, see Table S2); (iii) proteins differentially regulated between SD and SS (same cut-off parameters, see Table S3). The proteins common to at least two lists are presented in Table S4. Typical examples of differentially regulated proteins are shown in Figure 4, for spots number 2202, 1734, and 1869, respectively.

### 3.2. Effect of Senescence in Ctl Conditions

**Figure 5:**
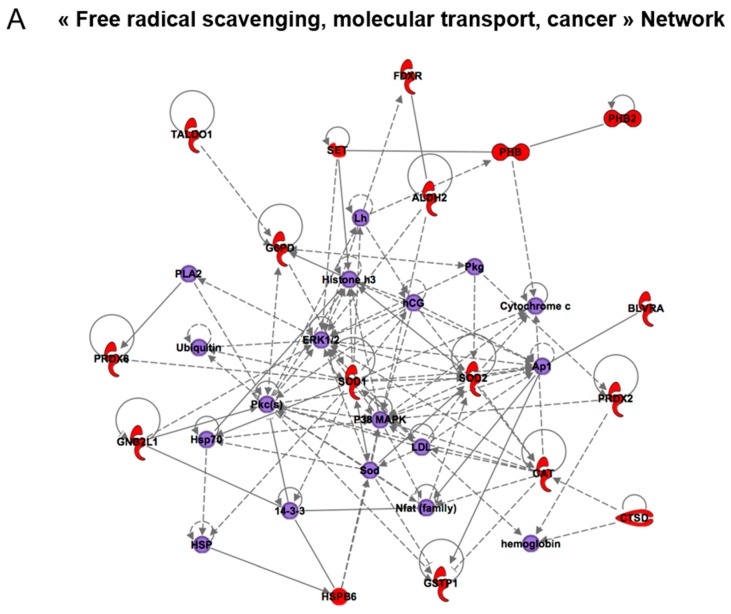

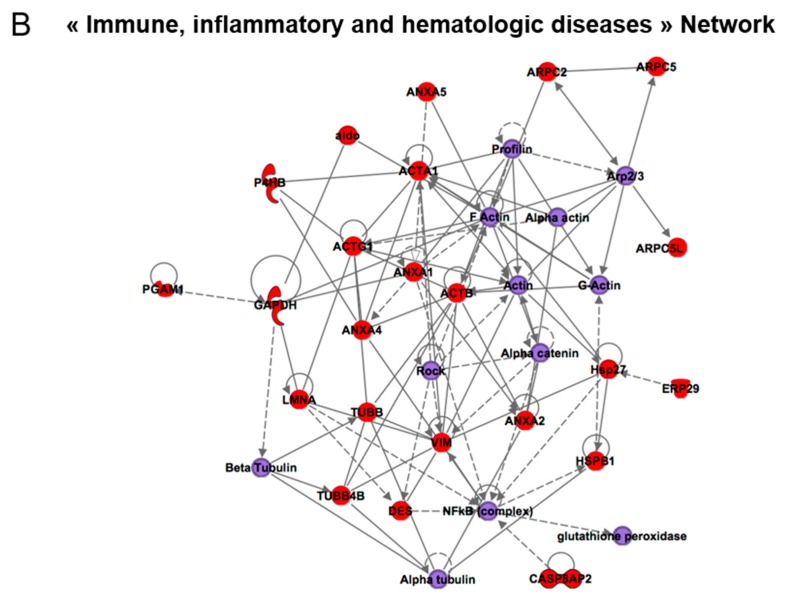
Illustration of the top networks identified by Ingenuity Pathway Analysis for the response to senescence (**A**) and selenium level variation (**B**). ACTA1: Actin, alpha skeletal muscle; ACTG1: Actin, cytoplasmic 2; ALDH2: Aldehyde dehydrogenase, mitochondrial; aldo: aldolase; ANXA1: Annexin A1; ANXA4: Annexin A4; ANXA5: Annexin A5; APA: Aminopeptidase A; Arp2/3: Actin-related protein 2/3; ARPC2: Actin-related protein 2/3 complex subunit 2; ARPC5: Actin-related protein 2/3 complex subunit 5; ARPC5L: Actin-related protein 2/3 complex subunit 5-like protein; BLVRA: Biliverdin reductase A; CASP8AP2: Caspase-8-associated protein 2; CAT: Catalase; CTSD: Cathepsin D; DES: Desmin; ERK1/2: Extracellular signal-regulated kinase 1/2; ERP29: Endoplasmic reticulum resident protein 29; FDXR: Ferredoxin reductase; G6PD: Glucose-6-phosphate 1-dehydrogenase; GAPDH: Glyceraldehyde-3-phosphate dehydrogenase; GNB2L1: Guanine nucleotide-binding protein subunit beta-2-like 1; GSTP1: Glutathione S-transferase P; hCG: Human chorionic gonadotropin; HSP: Heat shock protein; HSP27: Heat shock protein beta-1; HSPB1: Heat shock protein beta-1; HSPB6: Heat shock protein beta-6; LDL: Beta Lipoprotein; Lh: Light-harvesting complex; LMNA: Prelamin-A/C; Nfat: Nuclear factor of activated T-cells; NKkB: Nuclear factor NF-kappa-B; P38 MAPK: Mitogen-activated protein kinase 14; P4HB: Protein disulfide-isomerase; PBH: Prohibitin; PGAM1: Phosphoglycerate mutase 1; PHB2: Prohibitin-2; pkg: protein KINASE G; PLA2: Phospholipases A2; PRDX2: Peroxiredoxin-2; PRDX6: Peroxiredoxin-6; SET: Protein SET; SOD1: Superoxide dismutase [Cu-Zn]; SOD2: Superoxide dismutase [Mn], mitochondrial; TALDO1: Transaldolase; TUBB: Tubulin beta chain; TUBB4B: Tubulin beta-4B chain; VIM: Vimentin.

Many proteomic studies have investigated the phenotype of premature senescence, induced either by drug therapy (TIS) or by repeated exposure to mild oxidative stress (SIPS), but very few have investigated the replicative senescence of human diploid cells, such as WI-38 [22,30,31]. From a comparative study, it appeared that SIPS, TIS, and replicative senescence have distinct phenotypes but limited protein targets and pathways in common [22]. In this study, the authors found 50 spots with significant differences (±30% and *t*-test < 0.05) between young and senescent cells. Among these 50 spots, 12 were common with two different models of SIPS, suggesting that partially distinct mechanisms are involved. Unfortunately, in this study, the proteins were not identified by mass spectrometry. Here, our analysis—performed with slightly different parameters (±50% and *t*-test < 0.05)—allowed the detection of 43 spots, differentially regulated between young and senescent cell extracts, from which 71 proteins were identified by mass spectrometry (see Table 1 and Table S1). Interestingly, we found three times more proteins whose expression was stimulated in senescent compared to young cells than we did downregulated proteins. Among the most upregulated proteins, we found prohibitin-2 (PHB2), 78 kDa glucose-regulated protein (HSP5A), the cytoplasmic malate dehydrogenase (MDH1), heat shock cognate 71 kDa protein (HSPA8), and Vinculin (VCL), in spots 1546, 789, 1500, 761, and 754, respectively (see Figure 3 for spot location). In contrast, several proteins were found to be downregulated during replicative senescence, including the cytosolic superoxide dismutase (SOD1), the mitochondrial heat shock protein 75 kDa (TRAP1), the serine-threonine kinase receptor-associated protein (STRAP), and Vimentin (VIM) (Table 1 and Table S1). In principle, 2D DIGE allowed the detection of approximately 2500 spots which represent at maximum only 5 to 10% of the proteome, typically the most abundant proteins. It follows that most of the information regarding low abundance proteins is missing. To uncover the molecular pathways that are affected by replicative senescence of other potentially identifiable targets of this phenotype, we performed an Ingenuity^®^ Pathway Analysis (IPA^®^) on the list of significantly differentially expressed proteins (Table 2). This analysis proposed the three most relevant networks, which were (i) free radical scavenging, molecular transport, cancer; (ii) developmental disorders, and neurological and inherited diseases; and (iii) cancer, neurological disease, and cell signaling. The best-represented network in terms of number of members identified by mass spectrometry is shown in Figure 5A. These data suggest that the free radical scavenging pathway is a target of replicative senescence in WI-38 cells, making relevant the impact of selenium in slowing down the senescence phenotype by improving antioxidant status and redox homeostasis.

### 3.3. Effect of Selenium on Young and Presenescent Cells

**Figure 6:**
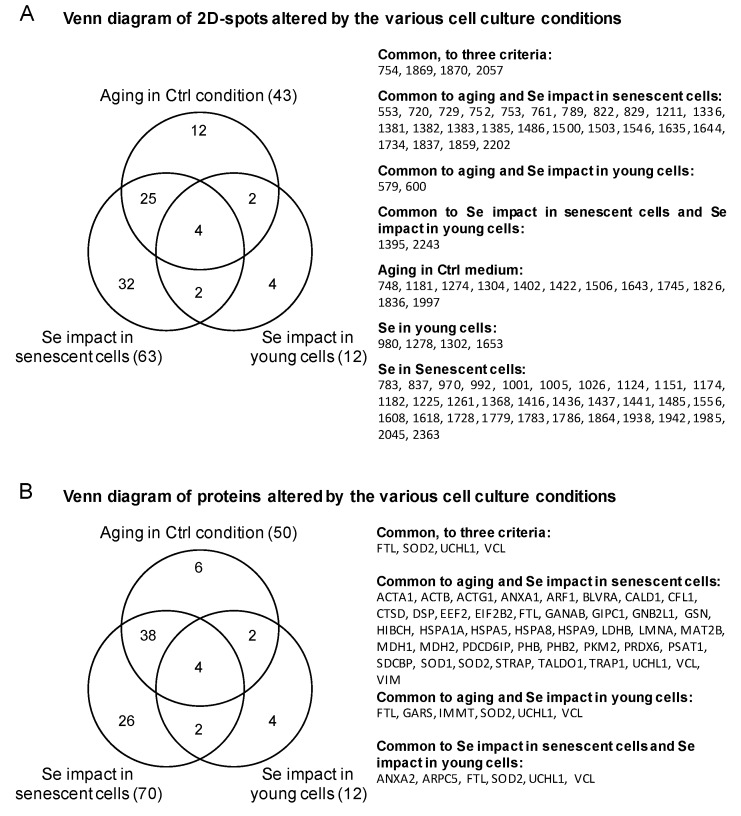
Venn diagram analyses for the differentially expressed spots (**A**) and identified proteins (**B**) in response to senescence and/or selenium level variation in young or senescent cells.

A similar analysis was performed to identify the proteins that were affected by selenium levels in either young or senescent cells. A cut-off of ±50% change in protein expression between Sup and Dpl with a *t*-test < 0.05 was applied. Spots 12 and 63 fulfilled these criteria in young and senescent conditions, respectively. The proteins identified by MS are listed in Table 1 and in Tables S2 and S3. Among these differentially expressed spots, 12 (young) and 70 (senescent) proteins were identified by mass spectrometry. It appears that selenium levels have much more impact in senescent than in young cells. In addition, the overlap of selenium-dependent variations between young and senescent cells is rather limited, as illustrated in the Venn diagrams in Figure 6. In the list of identified proteins regulated by selenium levels in senescent cells, we found 78 kDa glucose-regulated protein (HSPA5), Vinculin (VCL), Desmoplakin (DSP), Gelsolin (GSN), and Heat shock protein 70 kDa (HSPA1A) among the most upregulated proteins in Sup conditions. Among the most downregulated proteins in Sup conditions, we identified Ubiquitin carboxyl-terminal hydrolase isozyme L1 (UCHL1), Phosphoglycerate mutase 1 (PGAM1), Peroxiredoxin-6 (PRDX6), and Endoplasmic reticulum resident protein 29 (ERP29). An Ingenuity^®^ Pathway Analysis was then performed on the list of significantly differentially expressed proteins upon selenium variation (Table 3). This analysis proposed the three most relevant networks, namely: (i) immune, inflammatory, and hematologic diseases; (ii) cellular function and maintenance, energy production, lipid metabolism; and (iii) cancer, neurological diseases, and cell signaling. The first network, which is composed of 21 identified members, is shown in Figure 5B to illustrate the various connections of the network with associated groups or molecules.

### 3.4. Intersection between Selenium and Senescence

In order to determine whether the impact of selenium levels on the replicative potential of WI-38 fibroblasts involves similar pathways to regular senescence as suggested in [13], we investigated the overlap between the differentially expressed proteins listed in Tables S1–S3. As inferred by the Venn diagrams, the common targets encompassed 31 spots (Figure 6A), from which 44 proteins were identified by mass spectrometry (Figure 6B and Table S4). Our data show that 72% of the variation observed between young and senescent cells (31 spots over 43) are common with the response to selenium level variation. This value strongly suggests that the change of selenium levels in the culture affects predominantly molecular pathways linked to replicative senescence and may not be related to a premature senescence phenotype like TIS or SIPS. The link between selenium and senescence is illustrated by several selected examples in Figure 6, namely, spots 2202, 1734, and 1869, identified as Superoxide dismutase Cu-Zn (SOD1), Prohibitin-1 (PHB), and Ubiquitin carboxyl hydrolase terminal (UCHL1), respectively. Spot 1869 (UCHL1) is a common target of senescence, selenium in young cells, and selenium in senescent cells. As shown in Figure 4C and Table 1, this protein is upregulated by selenium depletion (+51% in young and +134% in senescent cells) and in response to senescence (+54%). Interestingly, selenium supplementation seemed to counteract this upregulation in senescent cells. It is tempting to speculate that UCHL1 and thence the connected pathway of proteasome protein degradation were affected immediately after selenium changes in the culture media, and that this effect lasted up to the senescence stage. Interestingly, the level of spot 1869 in SS conditions was almost identical to that in young cells (YC). On the other hand, spot 2202 (SOD1) exemplifies the opposite situation (Figure 4A), where a downregulation of the protein occurred during replicative senescence (−118%) and selenium deficiency (−174% in senescent cells). This effect was counteracted in senescent cells by selenium supplementation to recover an almost identical level as that of young cells (compare SS with YC conditions in Figure 4A). Interestingly, the downregulation of this spot seemed to also occur at early passages in Dpl medium, suggesting an early event that could initiate the senescence phenotype. Another kind of regulation is exemplified by spot 1734 (PHB, Figure 4B), which is upregulated (+76%) during the senescence process and by selenium deficiency in senescent cells (63%). Similar to UCHL1 and SOD1, the level of PHB in selenium-supplemented senescent cells (SS) almost reached that of the young conditions (YC), again suggesting a mechanism for the selenium-dependent slow-down of the senescence phenotype. It is important to bear in mind that protein content does not always correlate with enzyme activity. Most enzymes are subjected to PTMs that can alter catalytic activity. Such PTMs are often visible on 2D-Gel electrophoresis. It will be necessary to further characterize PTM changes in identified targets of selenium and/or senescence.

## 4. Discussion

Our work has consisted of further investigating the initial findings of [13], who reported an acceleration of replicative senescence in WI-38 cells due to selenium deficiency. However, selenium supplementation of the culture media was found to improve the replicative potential of WI-38 cells via the p21, p16, p53, and Rb pathways and to reduce senescence-associated markers (SABG, SAHF, telomere shortening). Here, we have used a proteomic approach to study whether this phenotype was relevant to mechanisms implied in premature senescence, induced by repeated stress (SIPS) or by therapy (TIS), or shared common features with the generic replicative senescence phenotype. The proteomics approach used here is based on separation of the cellular proteome in 2D-gels, fluorescent labelling of the proteins allowing a high sensitivity, and reliable data for inter-gel quantitative comparison. In this work, we detected 43 spots differentially and significantly regulated between young and senescent WI-38 cell extracts grown in Ctl conditions. From these spots, 71 proteins were identified by mass spectrometry (Q-TOF or Orbitrap). We compared this set of proteomic data to that obtained by growing WI-38 in various selenium-containing media for two passages or until they reached the senescence stage. We found that the 2D spots differentially expressed in response to replicative senescence variation share a 72% overlap with the spots of interest (31 spots over 43) identified in response to selenium level variation. Our data strongly suggest that selenium variation interferes with the generic senescence program but not with the premature senescence induced by exogenous stimuli. These data further confirm the strong interplay between selenium level and replicative senescence. This is in agreement with the finding that reactive oxygen species (ROS) are increased in senescent cells, together with a reduction of antioxidant defense and cell metabolism [4]. We posit a hypothesis stipulating that most of the physiological effect of selenium is due to the activity of selenoproteins involved in redox cellular homeostasis and intracellular ROS levels. Selenoproteins are indeed highly responsive to selenium variation following a gene-specific prioritization of selenium incorporation in selenocysteine, also referred to as the selenoprotein hierarchy [32]. This phenomenon has been described in WI-38 cells and found to be involved in transcriptional and translational regulatory mechanisms. To further understand the causal role of selenoproteins in replicative senescence, it will be crucial to follow the impact of gene inactivation of each member of this group of proteins in the acceleration of the senescence phenotype as has been done for several other markers of senescence. For example, the gene inactivation of SOD1, which we found to be downregulated in senescent WI-38 cells, leads to induction of the senescence phenotype [33]. Similar strategies could be employed for selenoproteins by targeting individual members or factors involved in their biogenesis [4]. To date, a single work has reported the impact of selenoprotein knockdown, in this case, SelenoH (also known as SelH), in human MRC-5 fibroblasts [34]. The authors describe a rapid enhancement of senescence-associated markers—including SABG ROS levels and cell autofluorescence—and a decrease in proliferative capacity. The fact that selenoproteins were not found in this proteomic analysis is rather surprising, since they were found to be highly sensitive to selenium variation in a previous study using this cell line. A reasonable explanation could be that the selenoproteome is present at trace levels within the overall proteome. Even the most abundant selenoproteins such as Gpx1 and Gpx4 seemed to be expressed below the detection limits of the CyDye strategy. We would suggest the use of a selenium-specific detection strategy such as (i) radioactive ^75^Se labelling, followed by autoradiography [35] or (ii) nonradioactive selenium labeling and detection by elemental mass spectrometry [36,37]. Alternatively, a state-of-the-art label-free proteomic strategy may also offer a better proteome recovery. Interestingly, glutathione peroxidases were found in the Ingenuity Pathway Analysis, as illustrated in Figure 5B.

## 5. Conclusions

Our present work further specifies the action of selenium in replicative senescence of human diploid cells. Our data, from a proteomic perspective, indicate that a significant proportion of the proteins altered by senescence are at least partially counteracted by selenium supplementation, which could explain the improvement of the replicative potential and the reduction of senescence-associated markers observed in our previous work [13]. This study opens the path to selective characterization of selenoproteins in the mechanism of replicative senescence and, more globally, the mechanism of aging.

## Figures and Tables

**Table 1:** List of proteins identified by tandem mass spectrometry (MS/MS) (either by quadrupole-time-of-flight (Q-TOF) or Orbitrap) from the coomassie blue-stained gel (presented in Figure 3).

Master Spot Number							Targets of Senescence		Targets of Selenium in Young Cells		Targets of Selenium in Senescent Cells	
	Protein Name	Gene Name	MS Ident	Score	Nb	Nb acc	Average Ratio (S/Y)	*p* Value	Average Ratio (Sup/Dpl)	*p* Value	Average Ratio (Sup/Dpl)	*p* Value
				Mascot	Peptides							
**Proteins Targets Common to Three Conditions**												
754	Vinculin	VCL	Orbitrap	452	15	P18206	2.40	0.01800	2.68	0.01800	3.06	0.00540
2057	Ferritin light chain	FTL	Orbitrap	422	21	P02792	1.91	0.00260	−1.78	0.00046	−3.05	0.00000
1870	Superoxide dismutase [Mn], mitochondrial	SOD2	Orbitrap	165	5	P04179	1.58	0.00016	−1.64	0.00057	−2.42	0.00086
1870	Ubiquitin carboxyl-terminal hydrolase isozyme L1	UCHL1	Orbitrap	197	12	P09936	1.58	0.00016	−1.64	0.00057	−2.42	0.00086
1869	Ubiquitin carboxyl-terminal hydrolase isozyme L1	UCHL1	Q-TOF	251	9	P09936	1.54	0.00010	−1.51	0.00013	−2.34	0.00003
**Proteins Targets Common to Two Conditions**												
600	Glycyl-tRNA synthetase	GARS	Q-TOF	88	4	P41250	1.90	0.00059	−2.09	0.00150		
600	Mitochondrial inner membrane protein	IMMT	Q-TOF	72	2	Q16891	1.90	0.00059	−2.09	0.00150		
579	Glycyl-tRNA synthetase	GARS	Orbitrap	1047	47	P41250	1.64	0.00300	−1.63	0.00130		
579	Mitochondrial inner membrane protein	IMMT	Orbitrap	1000	45	Q16891	1.64	0.00300	−1.63	0.00130		
579	Vinculin	VCL	Orbitrap	940	31	P18206	1.64	0.00300	−1.63	0.00130		
1546	Prohibitin-2	PHB2	Q-TOF	191	6	Q99623	4.48	0.00078			2.69	0.00140
789	78 kDa glucose-regulated protein	HSPA5	Orbitrap	660	22	P11021	2.67	0.00280			4.47	0.00073
1500	L-lactate dehydrogenase B chain	LDHB	Orbitrap	235	6	P07195	2.61	0.00029			2.33	0.00010
1500	Malate dehydrogenase, cytoplasmic	MDH1	Orbitrap	380	15	P40925	−2.61	0.00029			2.33	0.00010
761	Heat shock cognate 71 kDa protein	HSPA8	Orbitrap	557	20	P11142	2.60	0.00540			1.79	0.01200
761	Stress-70 protein, mitochondrial	HSPA9	Orbitrap	573	20	P38646	2.60	0.00540			1.79	0.01200
753	Gelsolin	GSN	Orbitrap	252	10	P06396	2.40	0.01100			2.80	0.00230
1385	Actin, cytoplasmic 1	ACTB	Orbitrap	184	6	P60709	2.33	0.00099			1.96	0.01500
1385	Translation initiation factor eIF-2B subunit beta	EIF2B2	Orbitrap	302	9	P49770	2.33	0.00099			1.96	0.01500
1382	Biliverdin reductase A	BLVRA	Q-TOF	97	5	P53004	2.27	0.00130			2.14	0.00510
753	Heat shock protein 70 kDa	HSPA1A	Orbitrap	252	7	P08107	2.21	0.01100			2.80	0.00230
752	Heat shock protein 70 kDa	HSPA1A	Orbitrap	687	24	P08107	2.21	0.01500			2.50	0.00300
1503	Desmoplakin	DSP	Orbitrap	198	10	P15924	2.21	0.00190			1.90	0.00095
1503	L-lactate dehydrogenase B chain	LDHB	Orbitrap	225	5	P07195	2.21	0.00190			1.90	0.00095
1503	Malate dehydrogenase, mitochondrial	MDH2	Orbitrap	172	6	P40926	2.21	0.00190			1.90	0.00095
1383	Elongation factor 2	EEF2	Orbitrap	260	11	P13639	2.15	0.00120			2.42	0.01200
1383	PDZ domain-containing protein GIPC1	GIPC1	Orbitrap	244	7	O14908	2.15	0.00120			2.42	0.01200
1383	Transaldolase	TALDO1	Orbitrap	164	5	P37837	2.15	0.00120			2.42	0.01200
1837	Peroxiredoxin-6	PRDX6	Q-TOF	214	11	P30041	2.04	0.00020			−1.54	0.01300
553	Neutral alpha-glucosidase	GANAB	Orbitrap	416	15	Q14697	1.85	0.00007			−1.64	0.00140
553	Programmed cell death 6-interacting protein	PDCD6IP	Orbitrap	482	18	Q8WUM4	1.85	0.00007			−1.64	0.00140
553	Caldesmon	CALD1	Q-TOF	365	20	Q05682	1.85	0.00007			−1.64	0.00140
553	Neutral alpha-glucosidase AB	GANAB	Q-TOF	422	20	Q14697	1.85	0.00007			−1.64	0.00140
1635	Annexin A1	ANXA1	Orbitrap	486	12	P04083	1.84	0.00096			−2.54	0.00460
1635	Guanine nucleotide-binding protein subunit beta-2-like 1	GNB2L1	Orbitrap	647	22	P63244	1.84	0.00096			−2.54	0.00460
1635	Syntenin-1	SDCBP	Orbitrap	747	22	O00560	1.84	0.00096			−2.54	0.00460
1211	Actin, cytoplasmic 2	ACTG1	Q-TOF	63	4	P63261	1.83	0.02500			1.81	0.00014
1734	Prohibitin	PHB	Q-TOF	249	10	P35232	1.76	0.00053			−1.63	0.00160
1859	Ubiquitin carboxyl-terminal hydrolase isozyme L1	UCHL1	Q-TOF	119	5	P09936	1.69	0.03500			−2.18	0.00023
1336	3-hydroxyisobutyryl-CoA hydrolase, mitochondrial	HIBCH	Orbitrap	181	5	Q6NVY1	1.62	0.00014			−1.69	0.01700
1336	Methionine adenosyltransferase 2 subunit beta	MAT2B	Orbitrap	186	4	Q9NZL9	1.62	0.00014			−1.69	0.01700
1336	Phosphoserine aminotransferase	PSAT1	Orbitrap	261	11	Q9Y617	1.62	0.00014			−1.69	0.01700
1644	Annexin A1	ANXA1	Q-TOF	198	5	P04083	1.52	0.00520			−1.91	0.00600
1644	Guanine nucleotide-binding protein subunit beta-2-like 1	GNB2L1	Q-TOF	110	4	P63244	1.52	0.00520			−1.91	0.00600
829	Pyruvate kinase isozymes M1/M2	PKM2	Q-TOF	299	14	P14618	−1.54	0.00200			1.70	0.00280
1486	Actin, alpha skeletal muscle	ACTA1	Q-TOF	259	5	P68133	−1.60	0.00190			1.61	0.00044
1486	Actin, cytoplasmic 2	ACTG1	Q-TOF	259	12	P63261	−1.60	0.00190			1.61	0.00044
822	Pyruvate kinase isozymes M1/M2	PKM2	Q-TOF	152	9	P14618	−1.78	0.00130			2.59	0.00069
1381	Serine-threonine kinase receptor-associated protein	STRAP	Orbitrap	667	17	Q9Y3F4	−1.87	0.00004			1.65	0.02300
1381	Vimentin	VIM	Orbitrap	269	13	P08670	−1.87	0.00004			1.65	0.02300
720	Heat shock protein 75 kDa, mitochondrial	TRAP1	Q-TOF	143	6	Q12931	−1.89	0.00056			2.23	0.00120
729	Heat shock protein 71 kDa	HSPA8	Orbitrap	702	24	P11142	−2.01	0.00046			2.54	0.00110
729	Prelamin-A/C	LMNA	Orbitrap	577	23	P02545	−2.01	0.00046			2.54	0.00110
729	Heat shock protein 75 kDa, mitochondrial	TRAP1	Orbitrap	783	32	Q12931	−2.01	0.00046			2.54	0.00110
2202	ADP-ribosylation factor 1	ARF1	Orbitrap	298	9	P00441	−2.18	0.00093			2.74	0.00160
2202	Cofilin-1	CFL1	Orbitrap	193	5	P23528	−2.18	0.00093			2.74	0.00160
2202	Superoxide dismutase [Cu-Zn]	SOD1	Orbitrap	399	13	P00441	−2.18	0.00093			2.74	0.00160
**Proteins Targets of One Condition**												
1836	Peroxiredoxin-6	PRDX6	Q-TOF	197	11	P30041	1.80	0.00001				
1826	Heat shock protein beta-1	HSPB1	Q-TOF	324	11	P04792	1.74	0.00031				
1506	26S proteasome non-ATPase regulatory subunit 14	PSMD14	Q-TOF	139	6	O00487	1.73	0.02200				
1745	Cathepsin D	CTSD	Q-TOF	195	8	P07339	1.68	0.00002				
1422	Reticulocalbin-1	RCN1	Q-TOF	432	19	Q15293	1.67	0.00027				
1422	Vimentin	VIM	Q-TOF	68	3	P08670	1.67	0.00027				
1643	Electron transfer flavoprotein subunit alpha, mitochondrial	ETFA	Orbitrap	1130	47	P13804	1.62	0.00035				
1181	Eukaryotic initiation factor 4A-I	EIF4A1	Q-TOF	138	5	P60842	−1.53	0.00300				
748	Pyruvate kinase isozymes M1/M2	PKM2	Q-TOF	236	7	P14618	−1.59	0.00074				
1304	Spermine synthase	SMS	Q-TOF	116	5	P52788	−1.62	0.00049				
1304	Vimentin	VIM	Q-TOF	83	5	P08670	−1.62	0.00049				
1402	Transaldolase	TALDO1	Q-TOF	279	7	P37837	−1.66	0.01100				
1274	Actin, cytoplasmic 2	ACTG1	Q-TOF	296	9	P63261	−1.76	0.00100				
1395	Annexin A2	ANXA2	Q-TOF	122	4	P07355			2.16	0.00300	2.13	0.00930
2243	Actin-related protein 2/3 complex subunit 5	ARPC5	Q-TOF	175	5	O15511			1.91	0.00230	1.57	0.02100
1653	Delta(3,5)-Delta(2,4)-dienoyl-CoA isomerase, mitochondrial	ECH1	Orbitrap	820	21	O15144			1.53	0.00001		
1278	Protein SET	SET	Q-TOF	116	3	Q01105			−1.54	0.00620		
980	Glucose-6-phosphate 1-dehydrogenase	G6PD	Q-TOF	279	14	P11413			−1.57	0.00000		
1302	Sialic acid synthase	NANS	Q-TOF	208	6	Q9NR45			−1.60	0.00000		
783	78 kDa glucose-regulated protein	HSPA5	Orbitrap	887	31	P11021					3.00	0.00100
2045	Desmoplakin	DSP	Q-TOF	504	18	P15924					2.87	0.00460
1124	Actin, cytoplasmic 2	ACTG1	Q-TOF	383	14	P63261					1.75	0.00380
1124	POTE ankyrin domain family member E	POTEE	Q-TOF	269	7	Q6S8J3					1.75	0.00380
2363	Actin-related protein 2/3 complex subunit 5-like protein	ARPC5L	Orbitrap	961	36	Q4R5P2					1.73	0.00860
2363	Heat shock protein beta-6	HSPB6	Orbitrap	554	28	O14558					1.73	0.00860
837	Catalase	CAT	Q-TOF	176	9	P04040					1.68	0.00940
837	Pyruvate kinase isozymes M1/M2	PKM2	Q-TOF	151	6	P14618					1.68	0.00940
1174	Fumarate hydratase, mitochondrial	FH	Q-TOF	244	5	P07954					1.64	0.03400
1783	Endoplasmic reticulum resident protein 29	ERP29	Q-TOF	238	7	P30040					1.55	0.05000
1864	Ubiquitin carboxyl-terminal hydrolase isozyme L1	UCHL1	Q-TOF	121	6	P09936					−1.50	0.02900
1786	Phosphoglycerate mutase 1	PGAM1	Q-TOF	65	3	P18669					−1.53	0.00009
1779	Endoplasmic reticulum resident protein 29	ERP29	Orbitrap	447	14	P30040					−1.55	0.01100
1779	Beta-hexosaminidase subunit beta	HEXB	Orbitrap	533	21	P07686					−1.55	0.01100
1779	Nicotinamide *N*-methyltransferase	NNMT	Orbitrap	415	19	P40261					−1.55	0.01100
1005	Tubulin beta chain	TUBB	Q-TOF	311	10	P07437					−1.56	0.00035
1416	Annexin A1	ANXA1	Q-TOF	343	13	P04083					−1.56	0.00500
1728	Cathepsin D	CTSD	Q-TOF	175	8	P07339					−1.56	0.00900
1261	Fructose-bisphosphate aldolase A	ALDOA	Q-TOF	196	8	P04075					−1.57	0.00380
1437	Glyceraldehyde-3-phosphate dehydrogenase	GAPDH	Q-TOF	222	7	P04406					−1.61	0.01100
1225	Isocitrate dehydrogenase [NADP] cytoplasmic	IDH1	Q-TOF	375	14	O75874					−1.63	0.02300
1026	Aldehyde dehydrogenase, mitochondrial	ALDH2	Q-TOF	185	5	P05091					−1.65	0.00004
1026	Xaa-Pro dipeptidase	PEPD	Q-TOF	184	5	P12955					−1.65	0.00004
1026	Tubulin beta-4B chain	TUBB4B	Q-TOF	140	6	P68371					−1.65	0.00004
1985	Peroxiredoxin-2	PRDX2	Q-TOF	201	10	P32119					−1.65	0.00380
970	Protein disulfide-isomerase A3	PDIA3	Q-TOF	409	19	P30101					−1.70	0.00200
970	Vimentin	VIM	Q-TOF	158	4	P08670					−1.70	0.00200
1618	Annexin A5	ANXA5	Q-TOF	175	11	P08758					−1.71	0.00002
1182	Desmin	DES	Q-TOF	415	18	P17661					−1.77	0.01400
1182	Vimentin	VIM	Q-TOF	415	18	P08670					−1.77	0.01400
1441	Glyceraldehyde-3-phosphate dehydrogenase	GAPDH	Q-TOF	246	6	P04406					−1.77	0.00650
1942	Glutathione S-transferase P	GSTP1	Q-TOF	329	10	P09211					−1.82	0.00094
1938	Glutathione S-transferase P	GSTP1	Orbitrap	1357	40	P09211					−1.85	0.00000
992	Vimentin	VIM	Orbitrap	2287	97	P08670					−1.87	0.00400
1368	Actin, cytoplasmic 1	ACTB	Q-TOF	215	8	P60709					−2.02	0.00056
1001	Tubulin beta chain	TUBB	Q-TOF	213	6	P07437					−2.03	0.00010
1151	Protein disulfide-isomerase	P4HB	Q-TOF	121	3	P07237					−2.04	0.00270
1151	Ribonuclease inhibitor	RNH1	Q-TOF	86	3	P13489					−2.04	0.00270
1151	Vimentin	VIM	Q-TOF	40	2	P08670					−2.04	0.00270
1556	l-lactate dehydrogenase B	LDHB	Orbitrap	234	7	P07195					−2.05	0.00310
1556	Inorganic pyrophosphatase	PPA1	Orbitrap	376	21	Q15181					−2.05	0.00310
1556	Tubulin beta-6 chain	TUBB6	Orbitrap	239	8	Q9BUF5					−2.05	0.00310
1436	Glyceraldehyde-3-phosphate dehydrogenase	GAPDH	Q-TOF	206	6	P04406					−2.07	0.00900
1608	Annexin A4	ANXA4	Q-TOF	481	28	P09525					−2.17	0.00011
1485	Annexin A1	ANXA1	Q-TOF	108	4	P04083					−2.26	0.00032
1485	Malate dehydrogenase, cytoplasmic	MDH1	Q-TOF	99	3	P40925					−2.26	0.00032

Nb: number; Sup/Dpl: Supplemented vs. Depleted.

**Table 2:** Ingenuity pathway analysis of functional networks affected by replicative senescence.

Network	Members	Molecules	Associated Groups or Molecules
Free radical scavenging, molecular transport, cancer	20	ACTA1, ACTG1, ANXA1, ANXA5, CASP8AP2, CTSD, FDXR, HSPB1, LMNA, PHB2, PHB, PRDX2, PRDX6, PSMD14, SMS, SOD1, SOD2, STRAP, TRAP1, VIM	14-3-3, 26S Proteasome, caspase, CD3, Cytochrome c, estrogen receptor, F actin, glutathione peroxidase, hemoglobin, Insulin, Lh, NFkB (complex), Rb, Ubiquitin
Developmental disorder, neurological and inherited diseases	13	BLVRA, EIF4A1, ETFA, GARS, GNB2L1, IMMT, PKM2, RAB11A, RCN1, TALDO1, TMEM132D, UCHL1, VSP13D	AkT, Ap1, CENPI, ERK1/2, FAM189B, FRY, FSH, Histone h3, Jnk, LEMD2, Mapk, NUDT6, P38 MAPK, PDGFB, PI3K (complex), Pkc(s), PTCD2, SLC9A6, SPRED3, UBC, USP35, USP40
Cancer, neurological disease and cell signaling	1	CEP104	NR2F6

**Table 3:** Ingenuity Pathway Analysis of functional networks affected by selenium in young or senescent cells.

Network	Members	Molecules	Associated Groups or Molecules
Immune, inflammatory and hematologic diseases	21	ACTA1, ACTB, ACTG1, ALDH2, ANXA1, ANXA2, ANXA4, ANXA5, DES, FDXR, GADPH, LMNA, P4HB, PHB2, PPA1, RNH1, STRAP, TUBB, TUBB4B, VIM	Actin, alpha actin, alpha tubulin, Ap1, Beta tubulin, CD3, estrogen receptor, F actin, FSH, Insulin, Lh, NFkB (complex), Profilin, Rock
Cellular Function and Maintenance, energy production, lipid metabolism	12	ALDOA, BLVRA, CAT, FH, IDH1, MDH1, PEPD, PKM2, POTEE/POTEF, PSMD14, TRAP1, VPS13D	26S proteasome, Akt, BLVRB, ERK1/2, Histone H4, IL22R1-IL10R2, INPP4B, Jnk, Jun-GABP, LEMD2, Mapk, N-arachidonylglycine, NUDT6, P38 MAPK, Pkc(s), PSME4, S100A16, SBSN, SLC26A6, SPRED3, TBCE, UBC, Ybx1-ps3
Cancer, neurological disease and cell signaling	1	CEP104	NR2F6

## Supplementary Materials

The following are available online at http://www.mdpi.com/2076-3921/7/1/19/s1, Table S1: Proteins that are differentially expressed between young (Y) and senescent (S) WI-38 grown in Ctl media; Table S2: Proteins that are differentially expressed between Sup and Dpl conditions in Young WI-38 cells; Table S3: Proteins that are differentially expressed between Sup and Dpl conditions in Senescent WI-38 cells; Table S4: List of proteins that are affected by senescence and by selenium (either in young or senescent cells).

## Abbreviations

ACTA1: Actin, alpha skeletal muscle; ACTB: Actin, cytoplasmic 1; ACTG1: Actin, cytoplasmic 2; AkT: RAC serine/threonine-protein kinase; ALDH2: Aldehyde dehydrogenase, mitochondrial; ALDOA: Fructose-bisphosphate aldolase A; ANXA1: Annexin A1; ANXA2: Annexin A2; ANXA4: Annexin A4; ANXA5: Annexin A5; Ap1: Activator protein 1; ARF1: ADP-ribosylation factor 1; ARPC5: Actin-related protein 2/3 complex subunit 5; BLVRA: Biliverdin reductase A; BLVRB: Biliverdin reductase B; CALD1: Caldesmon; CAT: Catalase; CD3: T-cell surface glycoprotein CD3; CENPI: Centromere protein I; CEP104: Centrosomal protein of 104 kDa; CFL1: Cofilin-1; CTSD: Cathepsin D; DES: Desmin; DSP: Desmoplakin; ECH1: Delta(3,5)-Delta(2,4)-dienoyl-CoA isomerase, mitochondrial; EEF2: Elongation factor 2; EIF2B2: Translation initiation factor eIF-2B subunit beta; EIF4A1: Eukaryotic initiation factor 4A-I; ERK1/2: Extracellular signal-regulated kinase 1/2; ERP29: Endoplasmic reticulum resident protein 29; ETFA: Electron transfer flavoprotein subunit alpha, mitochondrial; FAM189B: Family with sequence similarity 189, member B; FH: Fumarate hydratase, mitochondrial; FRY: Furry homolog; FSH: follicle- stimulating hormone; FTL: Ferritin light chain; G6PD: Glucose-6-phosphate 1-dehydrogenase; GANAB: Neutral alpha-glucosidase AB; GAPDH: Glyceraldehyde-3-phosphate dehydrogenase; GARS: Glycyl-tRNA synthetase; GARS: Glycyl-tRNA synthetase; GIPC1: PDZ domain-containing protein GIPC1; GNB2L1: Guanine nucleotide-binding protein subunit beta-2-like 1; GSN : Gelsolin; GSTP1: Glutathione S-transferase P; HEXB: Beta-hexosaminidase subunit beta; HIBCH: 3-hydroxyisobutyryl-CoA hydrolase, mitochondrial; HSPA1A: Heat shock protein 70 kDa; HSPA5: 78 kDa glucose-regulated protein; HSPA8: Heat shock protein 71 kDa; HSPA9: Stress-70 protein, mitochondrial; HSPB1: Heat shock protein beta-1; HSPB6: Heat shock protein beta-6; IDH1: Isocitrate dehydrogenase [NADP] cytoplasmic; IL22R1-IL10R2: Interleukin-22 receptor subunit alpha-1, Interleukin-10 receptor subunit beta; IMMT: Mitochondrial inner membrane protein; INPP4B: Type II inositol 3,4-bisphosphate 4-phosphatase; Jun-GABP: Jun, GA-binding protein subunit beta-1; LDHB: L-lactate dehydrogenase B; LEMD2: LEM (lamina-associated polypeptide-emerin-MAN1) domain containing 2; Lh complex: Light-harvesting complex; LMNA: Prelamin-A/C; Mapk: Mitogen-activated protein kinase; MAT2B: Methionine adenosyltransferase 2 subunit beta; MDH1: Malate dehydrogenase, cytoplasmic; MDH2: Malate dehydrogenase, mitochondrial; NANS: Sialic acid synthase; NFkB: Nuclear factor NF-kappa-B; NNMT: Nicotinamide N-methyltransferase; NR2F6: Nuclear receptor subfamily 2 group F member 6; NUDT6: Nucleoside diphosphate-linked moiety X motif 6; P38 MAPK: Mitogen-activated protein kinase 14; P4HB: Protein disulfide-isomerase; PDCD6IP: Programmed cell death 6-interacting protein; PDGF BB: Platelet-derived growth factor subunit B; PDIA3: Protein disulfide-isomerase A3; PEPD: Xaa-Pro dipeptidase; PGAM1: Phosphoglycerate mutase 1; PHB: Prohibitin; PHB2: Prohibitin-2; Pkc: Protein kinase C; PKM2: Pyruvate kinase isozymes M1/M2; POTEE: POTE ankyrin domain family member E; PPA1: Inorganic pyrophosphatase; PRDX2: Peroxiredoxin-2; PRDX6: Peroxiredoxin-6; PSAT1: Phosphoserine aminotransferase; PSMD14: 26S proteasome non-ATPase regulatory subunit 14; PSME4: Proteasome activator complex subunit 4; PTCD2: Pentatricopeptide repeat-containing protein 2, mitochondrial; Rb: Retinoblastoma; RCN1: Reticulocalbin-1; RNH1: Ribonuclease inhibitor; S100A16: Protein S100-A16; SBSN: Suprabasin; SDCBP: Syntenin-1; SET: Protein SET; SLC26A6: Solute carrier family 26 member 6; SLC9A6: Sodium/hydrogen exchanger 6; SMS: Spermine synthase; SOD1: Superoxide dismutase [Cu-Zn]; SOD2: Superoxide dismutase [Mn], mitochondrial; SPRED3: Sprouty-related, EVH1 domain-containing protein 3; STRAP: Serine-threonine kinase receptor-associated protein; TALDO1: Transaldolase; TBCE: Tubulin-specific chaperone E; TRAP1: Heat shock protein 75 kDa, mitochondrial; TUBB: Tubulin beta chain; TUBB4B: Tubulin beta-4B chain; TUBB6: Tubulin beta-6 chain; UBC: Ubiquitin C; UCHL1: Ubiquitin carboxyl-terminal hydrolase isozyme L1; USP35: Ubiquitin carboxyl-terminal hydrolase 35; USP40: Ubiquitin carboxyl-terminal hydrolase 40; VCL: Vinculin; VIM: Vimentin; Ybx1-ps3: Nuclease-sensitive element-binding protein 1-like

## References

[1] Papp L.V., Holmgren A., Khanna K.K. (2010). Selenium and selenoproteins in health and disease. Antioxid. Redox Signal..

[2] Latrèche L., Chavatte L. (2008). Selenium incorporation into selenoproteins, implications in human health. Metal Ions in Biol. Med. X.

[3] Whanger P.D. (2004). Selenium and its relationship to cancer: An update. Br. J. Nutr..

[4] Bulteau A.L., Chavatte L. (2015). Update on selenoprotein biosynthesis. Antioxid. Redox Signal..

[5] Gladyshev V.N., Arner E.S., Berry M.J., Brigelius-Flohe R., Bruford E.A., Burk R.F., Carlson B.A., Castellano S., Chavatte L., Conrad M. (2016). Selenoprotein gene nomenclature. J. Biol. Chem..

[6] Labunskyy V.M., Hatfield D.L., Gladyshev V.N. (2014). Selenoproteins: Molecular pathways and physiological roles. Physiol. Rev..

[7] Lobanov A.V., Hatfield D.L., Gladyshev V.N. (2009). Eukaryotic selenoproteins and selenoproteomes. Biochim. Biophys. Acta.

[8] Kryukov G.V., Castellano S., Novoselov S.V., Lobanov A.V., Zehtab O., Guigo R., Gladyshev V.N. (2003). Characterization of mammalian selenoproteomes. Science.

[9] Seeher S., Atassi T., Mahdi Y., Carlson B.A., Braun D., Wirth E.K., Klein M.O., Reix N., Miniard A.C., Schomburg L. (2014). Secisbp2 is essential for embryonic development and enhances selenoprotein expression. Antioxid. Redox Signal..

[10] Bosl M.R., Takaku K., Oshima M., Nishimura S., Taketo M.M. (1997). Early embryonic lethality caused by targeted disruption of the mouse selenocysteine tRNA gene (Trsp). Proc. Natl. Acad. Sci. USA.

[11] Touat-Hamici Z., Legrain Y., Sonet J., Bulteau A.-L., Chavatte L., Hatfield D.L., Schweizer S.U., Tsuji P.A., Gladyshev V.N. (2016). Alteration of selenoprotein expression during stress and in aging. Selenium: Its Molecular Biology and Role in Human Health.

[12] Touat-Hamici Z., Legrain Y., Bulteau A.L., Chavatte L. (2014). Selective up-regulation of human selenoproteins in response to oxidative stress. J. Biol. Chem..

[13] Legrain Y., Touat-Hamici Z., Chavatte L. (2014). Interplay between selenium levels, selenoprotein expression, and replicative senescence in wi-38 human fibroblasts. J. Biol. Chem..

[14] Papp L.V., Lu J., Striebel F., Kennedy D., Holmgren A., Khanna K.K. (2006). The redox state of secis binding protein 2 controls its localization and selenocysteine incorporation function. Mol. Cell. Biol..

[15] Childs B.G., Durik M., Baker D.J., van Deursen J.M. (2015). Cellular senescence in aging and age-related disease: From mechanisms to therapy. Nat. Med..

[16] Hayflick L., Moorhead P.S. (1961). The serial cultivation of human diploid cell strains. Exp. Cell Res..

[17] Campisi J., d’Adda di Fagagna F. (2007). Cellular senescence: When bad things happen to good cells. Nat. Rev. Mol. Cell Biol..

[18] Herbig U., Jobling W.A., Chen B.P., Chen D.J., Sedivy J.M. (2004). Telomere shortening triggers senescence of human cells through a pathway involving ATM, p53, and p21(CIP1), but not p16(INK4a). Mol. Cell.

[19] D’Adda di Fagagna F., Reaper P.M., Clay-Farrace L., Fiegler H., Carr P., Von Zglinicki T., Saretzki G., Carter N.P., Jackson S.P. (2003). A DNA damage checkpoint response in telomere-initiated senescence. Nature.

[20] Debacq-Chainiaux F., Borlon C., Pascal T., Royer V., Eliaers F., Ninane N., Carrard G., Friguet B., de Longueville F., Boffe S. (2005). Repeated exposure of human skin fibroblasts to UVB at subcytotoxic level triggers premature senescence through the TGF-beta1 signaling pathway. J. Cell Sci..

[21] Toussaint O., Royer V., Salmon M., Remacle J. (2002). Stress-induced premature senescence and tissue ageing. Biochem. Pharmacol..

[22] Dierick J.F., Eliaers F., Remacle J., Raes M., Fey S.J., Larsen P.M., Toussaint O. (2002). Stress-induced premature senescence and replicative senescence are different phenotypes, proteomic evidence. Biochem. Pharmacol..

[23] Flor A.C., Wolfgeher D., Wu D., Kron S.J. (2017). A signature of enhanced lipid metabolism, lipid peroxidation and aldehyde stress in therapy-induced senescence. Cell Death Discov..

[24] Wu M., Ye H., Shao C., Zheng X., Li Q., Wang L., Zhao M., Lu G., Chen B., Zhang J. (2017). Metabolomics-proteomics combined approach identifies differential metabolism-associated molecular events between senescence and apoptosis. J. Proteome Res..

[25] Olivieri O., Stanzial A.M., Girelli D., Trevisan M.T., Guarini P., Terzi M., Caffi S., Fontana F., Casaril M., Ferrari S. (1994). Selenium status, fatty acids, vitamins A and E, and aging: The nove study. Am. J. Clin. Nutr..

[26] Hornsby P.J., Harris S.E. (1987). Oxidative damage to DNA and replicative lifespan in cultured adrenocortical cells. Exp. Cell Res..

[27] Vacchina V., Dumont J. (2018). Total selenium quantification in biological samples by inductively coupled plasma mass spectrometry (ICP-MS). Methods Mol. Biol..

[28] Latreche L., Duhieu S., Touat-Hamici Z., Jean-Jean O., Chavatte L. (2012). The differential expression of glutathione peroxidase 1 and 4 depends on the nature of the SECIS element. RNA Biol..

[29] Redeker V., Bonnefoy J., Le Caer J.P., Pemberton S., Laprevote O., Melki R. (2010). A region within the C-terminal domain of Ure2p is shown to interact with the molecular chaperone Ssa1p by the use of cross-linkers and mass spectrometry. FEBS J..

[30] Dierick J.F., Kalume D.E., Wenders F., Salmon M., Dieu M., Raes M., Roepstorff P., Toussaint O. (2002). Identification of 30 protein species involved in replicative senescence and stress-induced premature senescence. FEBS Lett..

[31] Toussaint O., Medrano E.E., von Zglinicki T. (2000). Cellular and molecular mechanisms of stress-induced premature senescence (SIPS) of human diploid fibroblasts and melanocytes. Exp. Gerontol..

[32] Driscoll D.M., Copeland P.R. (2003). Mechanism and regulation of selenoprotein synthesis. Annu. Rev. Nutr..

[33] Blander G., de Oliveira R.M., Conboy C.M., Haigis M., Guarente L. (2003). Superoxide dismutase 1 knock-down induces senescence in human fibroblasts. J. Biol. Chem..

[34] Wu R.T., Cao L., Chen B.P., Cheng W.H. (2014). Selenoprotein H suppresses cellular senescence through genome maintenance and redox regulation. J. Biol. Chem..

[35] Yim S.H., Tobe R., Turanov A.A., Carlson B.A. (2018). Radioactive 75Se Labeling and Detection of Selenoproteins. Methods Mol. Biol..

[36] Sonet J., Mounicou S., Chavatte L. (2018). Nonradioactive isotopic labeling and tracing of selenoproteins in cultured cell lines. Methods Mol. Biol..

[37] Sonet J., Mounicou S., Chavatte L. (2018). Detection of selenoproteins by laser ablation inductively coupled plasma mass spectrometry (LA-ICP MS) in immobilized pH gradient (IPG) strips. Methods Mol. Biol..

